# Supplementary material for: Broadband highly directive 3D nanophotonic lenses
Source: Nat Commun. 2018 Nov 9;9:4742. doi: 10.1038/s41467-018-07104-1 (PMC6226513; doi:10.1038/s41467-018-07104-1)
Supplement: Supplementary file 1 — Supplementary Information [file 41467_2018_7104_MOESM1_ESM.pdf]

Supplemental Information
for
Broadband Highly Directive 3D Nanophotonic Lenses

Eric Johlin *et al.*

Supplementary Note 1

An evolutionary algorithm is used to optimize the 3D nanolens structures, and operates based on the following pseudocode:

```
procedure EVOLUTIONARY OPTIMIZATION PSEUDOCODE
    Generation X_0: Initialize random population of structures
    Calculate Directivity(X_0)
    Save Z best Individuals of X_0
    for each Generation X ∈ A do
        for each Individual y ∈ X  do
            function RANDOMIZE BETWEEN:
                - Create random structure
                - Breed 2 best structures
                - Breed 1 best structures and 1 random structure          ▷ Mutate structure
            end function
            Smooth raw structure (anisotropic convolution)              ▷ remove small features
            Compute Fitness(y) for smoothed structure
            if Fitness(y) > min(Fitness(Z)) then                ▷ where Z ≡ set of best structures
                y ∈ Z                                        ▷ Replace worst structure in Z with y
            end if
        end for
        Remove 20% of Z                                                   ▷ Chance to clear out
        If no improvement for 4 generations, leave loop
    end for
end procedure
```

The structures are defined as rings on a discretized grid of 40x40 matrix elements (i.e. pixels), corresponding to the rectangular cross-sectional regions of the rings. The presence of a ring denotes a refractive index of 1.4, whereas the absence is 1.0. The optimization is binary in that no intermediate refractive indices are allowed.

The algorithm is initialized by creating a starting population (X_0) of 30 fully random structures (y). These are composed of a random number of rings (average of 20) with cross-sectional sizes ranging from 5 to 20 pixels. After a structure is created, it is convolved with a smoothing matrix approximating the point spread function of the lithography tool ($\sim 200 \times 200 \times 600$ nm), and the simulation is run (see Supplemental Information Note II). The 6 structures with the highest fitness (photoluminescence-weighted absorption in the nanowire at normal incidence, used here as a proxy for the spatially-averaged directivity along the nanowire) are stored in the breeding pool (Z). For all generations following the

first, when the population (X) of 30 new structures is produced, each structure has an equal chance of three possibilities: to create a new random structure (as in the first generation); to breed two structures from the breeding pool; or to breed one structure with a random structure (*i.e.* mutate a structure).

The breeding is performed by overlaying the two structural matricies to be bred, maintaining agreeing regions, and using a coarse 4x4 matrix to determine areas of differences. The specific size of the 4x4 matrix is randomized each time to avoid preferential transition lines from forming, and the coarse size is used to reduce the creation of unwritably small features. When the fitnesses of the new structures are computed, if a structure exhibits a fitness greater than that of the lowest member of the breeding pool, that new member takes the place of the previous lowest member. It however is not used for breeding until the subsequent generation. Additionally, at the end of each generation, there is a 20% chance for each (non-new) member of Z to be removed from the pool, leaving an open spot. This is performed to prevent stagnation of the pool, and helps to avoid the algorithm becoming stuck in local minima.

The algorithm proceeds until either a large number of generations has passed (40; to avoid divergence) or a change in the maximum fitness of greater than 1% has not been achieved for 3 subsequent generations (this often occurs within ~25 generations).

Supplementary Note 2

Simulations are performed using FDTD Solutions (Lumerical Inc.). Reciprocity is leveraged in simulating the emission of the nanowire, in that the simulation is performed by calculating the absorption cross section of the nanowire-lens system under plane wave illumination. This is done to account for the fact that emission/absorption events can occur at any position along the wire (e.g. the entire volume of the wire is a finite sized emitter), as opposed to the simpler case where the emission from a point emitter can be computed in a single simulation. It is seen that the absorption cross section the nanowire is increased by a factor of approximately 32 when the nanolens is applied.

After the optimization is completed, the best performing structures are inspected, and any structures with features that would be difficult to fabricate are ignored. The best producible structures are then analyzed under a series of simulations placing point dipole emitters of the three cardinal orientations at the center of the nanowire, and computing the electric and magnetic field components on a box around the structure for each. This data is then processed using a freely available software package[1] to compute the angular distribution of emission, accounting for the interface due to the presence of the substrate. The transformation is performed for each wavelength computed, with example 2D slices of the emission profiles shown in Supplementary Figure 1.

Furthermore, it is important to ensure that the designed structure is sufficiently robust against small deviations in fabrication. In Supplementary Figure 2 we calculate the influence of displacement of the nanolens into the substrate on the expected directivity, representing the sensitivity to misalignment in the vertical direction (failure to write the nanolens perfect at the interface). We see little change in the expected response for displacements as large as 200 nm into the substrate, indicating a significant range of acceptable positions at which the writing could occur. After this point the response falls off linearly to approximately one full micron into the substrate, at which point the lens structure would be significantly modified.

Once the spectral directivity for center-point emission is calculated, displaced dipole

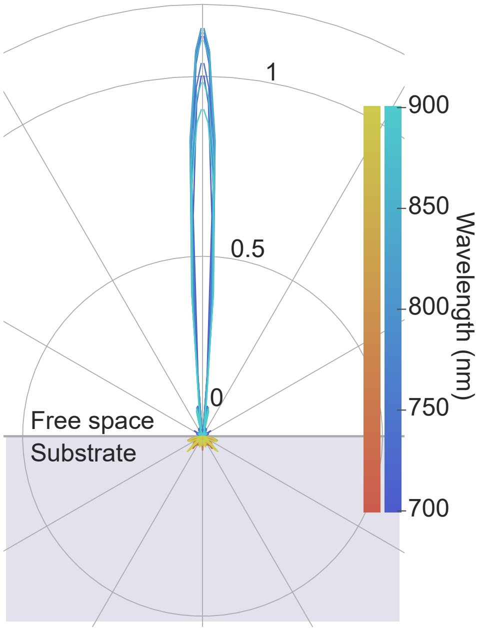

Supplementary Figure 1: Broadband directivity. Simulated point-emitter spectral response of nanowire-nanolens system, showing high directivity for all considered wavelengths. The intensity of emission is shown by the radial distance to the curve. Emission into the upper hemisphere (free space) is denoted in the blue scale, while emission into the substrate is orange.

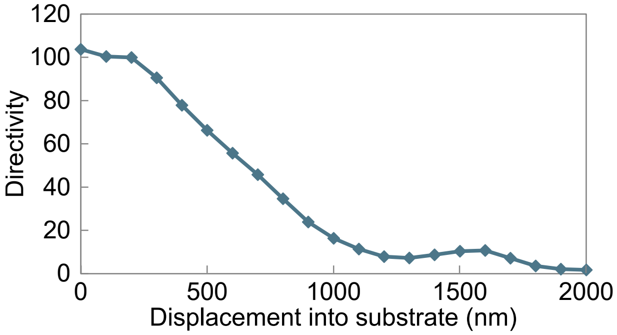

Supplementary Figure 2: Robustness to vertical misalignment. Simulated directivity response of the designed nanolens structure when vertically misaligned into the substrate.

simulations are performed along distances from the center to the end of the nanowire, in 10 nm increments. The projected far field from a single monitoring plane above the lens structure is recorded. The plane is chosen to be sufficiently large that it contains emission from all angles contained within the numerical aperture (NA) of the experimental objective (0.9 NA). Due to the proximity of the plane, this will not necessarily capture all light that the objective would see, but serves as a reasonable approximation for the imaged emission, and precludes additional expensive transformation calculations from being performed.

The simulated emission from all points along the nanowire can then be combined into arbitrary patterns. This allows fitting of the nanowire profile to the lens position to estimate

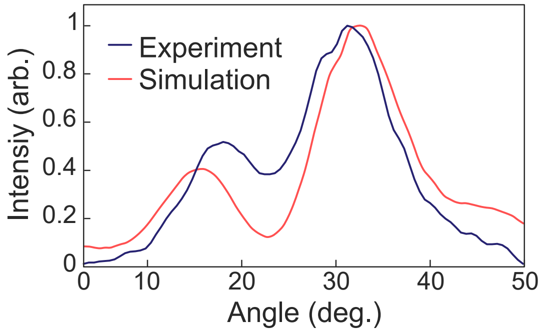

Supplementary Figure 3: Structure of beaming spot. Line scan of beaming peak and side-lobe, comparing the measured profile to the simulated system, showing agreement in the two-lobed features between the experiment (black) and simulation (red).

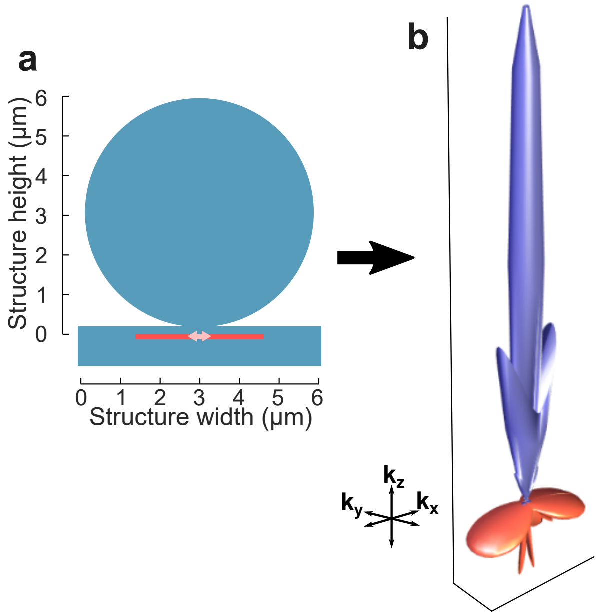

Supplementary Figure 4: Microsphere-nanowire system. **a** Schematic, showing the dielectric microsphere and substrate (blue), and GaAs nanowire (red) with emission at the center of the nanowire (pink arrow). **b** Relative emission diagram, for comparison to the nanolens discussed in this work, showing approximately 1/3 of the directivity of the algorithmically-designed lens. Red and blue regions correspond to emission into, and out of the substrate, respectively.

the misalignment between the lens and nanowire. The fitting is performed qualitatively, but the accuracy of the fit can still be assessed. Supplementary Figure 3 shows a comparative line profile across the major and minor peaks present in both the simulation and experimental measurements (horizontal slice through the denoted maximum in Fig. 4 **b** and **c** of the main text, showing reasonable agreement between the two profiles. The produced profiles here are then treated in the same manner as the experimental measurements (albeit with a zero background) for computation of the simulated directivity for the finite emitter, and misaligned nanolens systems.

Simulations of spherical lenses are also performed to compare the algorithmically-designed lens to traditional lens designs. The position of a sphere is optimized over the same nanowire-centered dipole as the point-emitter directivity is calculated for the nanolens investigated in this work. The optimization allows the position of the sphere to be placed anywhere from halfway into the substrate (hemispherical lens) to directly on top of the substrate. It is found that the highest achievable directivity in such a system is 36.6, compared to the 101 achieved with a algorithmically-optimized lens of the same footprint. The 3D emission pattern of the microsphere is shown in Supplementary Figure 4, and can be compared to that of Fig. 2**d** of the main text.

Supplementary Note 3

The size of the emitting region is determined from real-space microscopy images of the nanowire fluorescence, shown in Supplementary Figure 5**a**. Profiles along the short and long axis of the emission (corresponding to the short and long axis of the nanowire) are measured and shown in Supplementary Figure 5**b**. These profiles are fit to Gaussian functions, giving standard deviations of 580 and 340 nm, respectively. The short axis profile is used as the measurement point response function, and deconvolved from the long axis profile, yielding an estimated emitting region standard deviation of 240 nm.

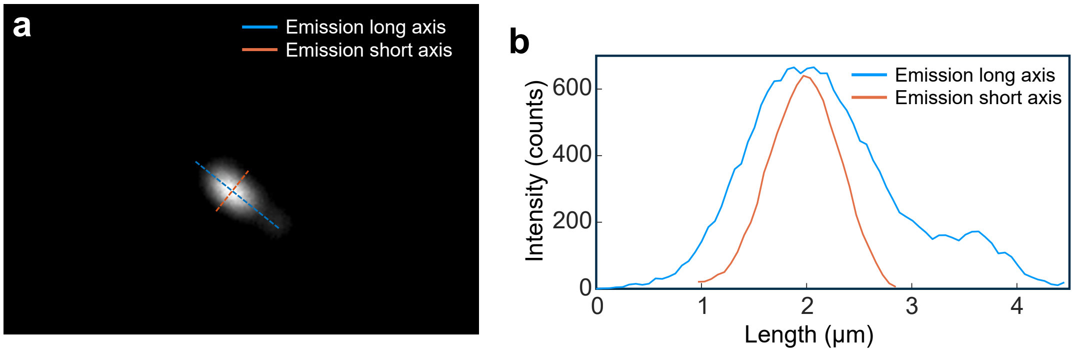

Supplementary Figure 5: Real-space nanowire emission. **a** Microscope image of nanowire photoluminescence, with two indicated profiles along the long and short axis of the point spread function, as depicted in **b**. Scale bar is 3 μm.

The observable region of the back-focal plane is initially fit using emission from bright nanowire clusters, allowing accurate definition of the maximum NA of the measurement. The experimental measurements are then processed by first subtracting the background images from the nanowire measured data and removing 12 "dead" pixels from the camera data. A region away from the NA is then analyzed to determine the detector noise, establishing a background level and variance for the measurements.

The data within the NA is then stepped through, calculating the radial distance to each pixel, and creating a dataset of the mean radial response of the image. The maximum within the NA is also determined as an average of the pixel in question and the 8 surrounding pixels, to prevent single-pixel noise events from being recorded as a spurious peak. The radial response is weighted by a projection to a spherical section, and the partial directivity is computed for the angular space within the NA. The radial response, along with the

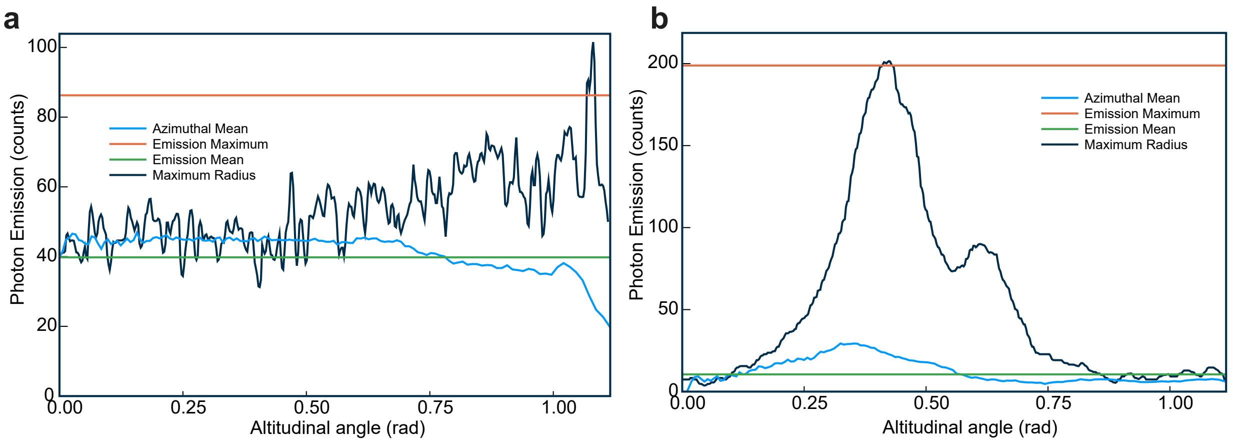

Supplementary Figure 6: Line profiles of the emission. Mean response, as well as the single azmiuthal angle containing the maximum emission, with respect to the zenithal angle (equivalent to the radial distance in Fig. 4 of the main text). The total mean and maximum (with 9 pixel average included) are shown as horizontal lines as well. **a** Bare nanowire emission, remaining fairly uniform over the entire observable range. The increased emission maximum is likely due largely to both noise and systematic error in the system, but cannot be reasonably excluded. **b** Nanowire-nanolens system, showing a clear peak in the radial response due to the off-center maximum due the strong beaming.

peak and mean are shown for both the bare nanowire and nanowire-nanolens system in Supplementary Figure 6. The averaging over 9 pixels in the calculation of the emission maximum (red) is responsible for it being somewhat below that of the peak in the azimuth containing the overall maximum value (black).

To compute the full directivity, the average emission in the 25% furthest region within the NA is extrapolated to the remaining unobservable region of angular space, assuming the emission follows a Lambertian profile, thus providing the upper-hemispherical emission. This integrated upper-hemisphere emission is then weighted by the simulated ratio of emission into the substrate, and used for the lower hemispherical emission. The ratio of the observed maximum, and the mean emission into the full sphere allows the full directivity to thus be computed.

Supplementary References

[1] Yang, J., Hugonin, J. P. & Lalanne, P. Near-to-Far Field Transformations for Radiative and Guided Waves. *ACS Photonics* **3**, 395–402 (2016).
