# Supplementary material for: Broadband highly directive 3D nanophotonic lenses
Source: Nat Commun. 2018 Nov 9;9:4742. doi: 10.1038/s41467-018-07104-1 (PMC6226513; doi:10.1038/s41467-018-07104-1)
Supplement: Supplementary file 3 — Description of Additional Supplementary Files [file 41467_2018_7104_MOESM3_ESM.pdf]

Description of Additional Supplementary Files

File Name: Supplementary Movie 1
Description: Video of simulated propagation of a light pulse through the nanolens structure, visualized as the real part of the electric field component perpendicular to the screen, as portrayed in snapshots in Fig. 2e of the main text.

File Name: Supplementary Software 1
Description: Setup for writing the nanolens structure investigated here, requires the coordinates file in Supplementary Software 2

File Name: Supplementary Software 2
Description: Coordinates for the nanolens structure investigated here, used by the setup file in Supplementary Software 1.
